# Improving Growth and Productivity of Oleiferous Brassicas under Changing Environment: Significance of Nitrogen and Sulphur Nutrition, and Underlying Mechanisms

**DOI:** 10.1100/2012/657808

**Published:** 2012-05-01

**Authors:** Naser A. Anjum, Sarvajeet S. Gill, Shahid Umar, Iqbal Ahmad, Armando C. Duarte, Eduarda Pereira

**Affiliations:** ^1^Centre for Environmental and Marine Studies (CESAM), Department of Chemistry, University of Aveiro, 3810-193 Aveiro, Portugal; ^2^The International Centre for Genetic Engineering and Biotechnology (ICGEB), Aruna Asaf Ali Marg, New Delhi 110067, India; ^3^Stress Physiology and Molecular Biology Lab, Centre for Biotechnology, MD University, Rohtak, India; ^4^Department of Botany, Faculty of Science, Hamdard University, New Delhi 110062, India

## Abstract

Mineral nutrients are the integral part of the agricultural systems. Among important plant nutrients, nitrogen (N) and sulphur (S) are known essential elements for growth, development, and various physiological functions in plants. Oleiferous brassicas (rapeseed and mustard) require higher amounts of S in addition to N for optimum growth and yield. Therefore, balancing S-N fertilization, optimization of nutrient replenishment, minimization of nutrient losses to the environment, and the concept of coordination in action between S and N could be a significant strategy for improvement of growth and productivity of oleiferous brassicas. Additionally, positive interaction between S and N has been reported to be beneficial for various aspects of oilseed brassicas. The current paper updates readers on the significance of N and S for the improvement of plant growth, development, and productivity in detail. In addition, S-N nutrition-mediated control of major plant antioxidant defense system components involved in the removal and/or metabolism of stress-induced/generated reactive oxygen species in plants (hence, the control of plant growth, development, and productivity) has been overviewed.

## 1. Introduction

Oleiferous brassicas (rapeseed and mustard) are among important food crops in Asia. In India, the oilseeds form the second largest agricultural commodity where among nine annual oilseed crops grown, the oleiferous brassicas are the major provider of edible oil to a major proportion of population. Moreover, India contributes about 30% and 20% in world acreage and production, respectively, of oilseed brassicas. It is one of the best edible oils available, having lowest amount of saturated fats as compared to other vegetable oils, and provides both essential fatty acids and also the animal feed through oil-free meal rich in protein having well-balanced aminogram and equally having potential for the purpose of biofuel [1–4].

Rapeseed and mustard are important species of Brassica grown as oilseeds. These have remained one of the major sources of edible for centuries. Presently, five Brassica species are cultivated as field crops. Among them, existence of “Sarson” (*B. campestris*), “Raya” (*B. juncea*), and “Taramira” (*Eruca sativa*) goes back to centuries. Introduction of Gobhi sarson (*B. napus*) is rather recent and its cultivation as a seed crop is confined only in few areas. According to FAO statistics [5], the production of rapeseed stands second after soybeans. The production of rapeseed was estimated 36.61, 46.17, and 46.41 metric tons during the years 2003, 2004, and 2005, respectively. Moreover, the rapeseed cultivation area in world was estimated 27.4, 29.7, and 30.3 million ha during the years 2006, 2007, and 2008, respectively. Additionally, average seed yield of rapeseed in world was noted 1.75, 1.73, and 1.91 tons ha^−1^ during the years 2006, 2007, and 2008 respectively. In view of increasing population and improved and increased standard of living of the people in 21st century, the requirement of fats and oils is bound to go high. To meet the minimal nutritional requirement of fats and oils (12 kg capita^−1^ year^−1^) for food, feed and other industries, and to earn sizable foreign exchange through export of seed meal, oil and value added products, nearly 24 million tonnes of mustard oilseed would be required by 2020 AD [6, 7].

Since last few decades, the growth, development, and productivity of oleiferous brassicas have been hampered due to a number of factors including the unbalanced plant mineral nutrients in soils. In fact, the extra pressure on the limited land resources and use of high yielding varieties to feed rapidly increasing population have led to the present scenario of shortage of important plant mineral nutrients in major soils on the globe. The deficiency of soil S in the agricultural soils has been reported frequently over the past several years [8–10]. Sulphur availability has been decreasing also in many areas of Europe during the last two decades [11–13]. However, among different regions, Asia has the highest S fertilizer requirement. In Asia, India and China alone currently account for about 60% of the total estimated deficit. Continuous mining of S from soils has led to widespread S deficiency and negative soil budget [14].

### 1.1. Sulphur and Nitrogen in relation to Oleiferous Brassicas

Sulphur (S) is one of the six macronutrients needed for proper plant development. The S requirement by plants varies with the developmental stage and with species whereas its concentration in plants varies between 0.1 and 1.5% of dry weight. Even if sulphur is only 3% to 5% as abundant as nitrogen (N) in plants, it plays essential roles in various important mechanisms such as Fe/S clusters in enzymes, vitamin cofactors, glutathione (GSH) in redox homeostasis, and detoxification of xenobiotics [9, 10, 15–17]. The reduced S incorporated in cysteine (Cys) and methionine (Met) amino acids plays essential roles in catalytic centers and disulfide bridges of proteins [18]. Additionally, Nitrogen (N) and S are necessary for the synthesis of amino acids, proteins, and various other cellular components, including thiol compounds and the so-called secondary sulphur compounds, which have a significant bearing on protection of plants against stress and pests.

It is pertinent to mention here that oleiferous brassicas (Brassicaceae) have greater S requirements than other large crop species such as wheat or maize and, therefore, are particularly sensitive to S deficiency because of their high demand for S [17, 19–22]. For example, the production of 1 tonne of rape seeds requires 16 kg of S [23, 24], compared with 2–3 kg for each tonne of grain in *Triticum aestivum* [13]. Hence, S deficiency in the soil, where these crops are raised, is considered as a major factor responsible for both low seed quality [25, 26] and yield by 40% [8]. Previously, sufficient S to meet crop requirements was obtained from the frequent incidental additions of S to soils when N and P fertilizers, such as ammonium sulfate and single superphosphate, were applied. S deficiencies can be the result of a combination of processes such as the replacement of traditional S-containing fertilizers with high-analysis N and P fertilizers containing little or no S [13, 27], while a massive decrease of S inputs from atmospheric deposition has been recorded during the last three decades. Moreover, it can also be suggested that the S requirements of many crops have increased as a result of intensive agriculture and optimization during plant breeding programmes, and yields of agricultural crops have increased markedly and, in some cases, more than doubled, resulting in increased removal of nutrients including S from soils [8, 14, 28].

Another, plant mineral nutrient N has been a critical element for plant growth, and plant response to added N has proven to be a valuable agronomic practice since time immemorial. Nitrogen is an integral component of amino nucleic acids, proteins, nucleotides, chlorophyll, chromosomes, genes, ribosomes and is also a constituent of all enzymes. This wide range of different nitrogen containing plant compounds explains the important role of nitrogen for plant growth. The nitrogen supply of oilseed rape is of central importance to ensure high yields. As oleiferous brassicas are heavy users of N, and available N is the most limiting source in many areas of the world [29, 30], therefore, mineral N fertilization is a crucial factor in oilseed rape production [28, 31, 32], and because of low harvest index of oleiferous brassicas high rates of N fertilizer are usually applied to this crop in order to obtain maximum seed yield [33] in diverse and contradicting conditions [34–38]. However, fertilizer N requirements can differ very much according to soil type, climate, management practice, timing, source, and rate of N application, cultivars, and so forth [39]. Uptake of N by oilseed rape crops is very high and in total may be over 250 kg N/ha [40, 41]. Moreover, S requirement and metabolism in plants including oleiferous brassicas are closely related to N nutrition [42, 43], and N metabolism is also strongly affected by the S status of the plant [43–45]. The assimilatory pathways of S and N have been considered functionally convergent and well coordinated as the availability of one element regulates the other [42, 46] and that C assimilation pathway is closely linked to nitrate assimilation in plants [21]. Moreover, Fismes et al. [47] have shown using field-grown oilseed rape that S deficiency can reduce nitrogen use efficiency (NUE: ratio of harvested N to N fertilization) and that N deficiency can also reduce sulphur use efficiency (SUE). Additionally, positive interaction between S and N has been reported to be beneficial for various aspects of oilseed brassicas including tolerance to various stress factors.

The current paper updates readers on the significance of N and S for the improvement of plant growth, development, and productivity in detail in the light of current literature. In addition, S-N nutrition-mediated control of major plant antioxidant defense system components involved in the removal and/or metabolism of stress-induced/generated reactive oxygen species in plants (hence, the control of plant growth, development, and productivity) has been overviewed.

## 2. Growth, Photosynthetic Functions and Seed/Oil Yield and Quality in Oleiferous Brassicas and Sulphur Nutrition

### 2.1. Growth and Photosynthetic Functions

The availability of S has been shown to influence the growth and photosynthetic functions to a great extent in oilseed brassicas [17, 21, 48–51]. According to Blake-Kalff et al. [50], developing leaves first exhibit the symptoms of S deficiency. Moreover, continuous S deficiency can lead to slower growth and fewer leaves in the later stages of oilseed rape development. Whereas, young leaves can be chlorotic and exhibit reduced photosynthetic activity as well. High S fertilization has been shown to increase RuBP, chlorophyll, and protein contents in fully expanded upper leaves of *B. juncea* (mustard) and *B. campestris*, which implies a better photosynthetic activity in comparison with plants grown without S [51]. As RuBP contains 120 cysteines and 168 methionines per molecule [48], therefore it seems to be an obvious target for mobilization when S amino acid synthesis is restricted by S deficiency [51]. Additionally, any decrease of Rubisco affects the photosynthesis rate, and the decline of chlorophyll also contributes to the breakdown of photosynthesis under S-deficient condition. Blake-Kalff et al. [50] have reported the degradation of chlorophyll in oilseed rape, particularly in the youngest leaves of plants grown on nutrient solution containing no S and high N, but not in leaves of plants grown on no S and low N. Moreover, authors also observed that when sulphate is removed from the nutrient solution, the concentration of glutathione (GSH, a low molecular weight, water-soluble, S-containing nonprotein thiol compound which functions in protection of plants against varied environmental stresses, De Kok et al. [52]) decreased rapidly in the middle and youngest leaves. However, the uptake of sulphate and its subsequent distribution to the leaves have been shown being closely regulated in response to demand *namely*, new developing leaves are strong S sinks, but show a net loss of S after full expansion [49, 50].

### 2.2. Seed/Oil Yield and Quality

Sulphur deficiency has been reported to influence the lipid and protein composition of seeds thus impacting nutritional quality [10, 53–57]. Ahmad and Abdin [21] studied the changes in the contents of lipid, RNA, and fatty acids in the developing seeds of *B. campestris* cultivar (cv.). Pusa Gold grown with or without S. Moreover, in +S treatments, authors applied S either as a single dose or the same dose was split in two or three portions. Authors observed rapid accumulation of lipids started at 7 days after flowering and continued until 35 days after flowering. Additionally, the lipid content in the seeds from the initial stage was found increased with S application, and the maximum increase was observed, when S was applied in three portions. Authors noticed a positive strong corelation between S and lipid content in the seeds. In addition, authors observed increase in the oleic acid (18 : 1) content but decrease in the erucic acid (22 : 1) content over other treatments, and authors argued that this may lead to a reduced 22 : 1 : 18 : 1 ratio and thus, improve the quality of oil. Moreover, the ratio of erucic acid to oleic acid (22 : 1 : 18 : 1) was found closely related to the N : S ratio in the seeds. Reports are available recommending the use of 30 and 60 kg ha^−1^ of S fertilizer for obtaining maximum yield [10, 19, 47, 53, 58]. Grant et al. [54] conducted field studies in Manitoba, Saskatchewan, and Alberta over 3 yr to evaluate immediate and residual effects of source, timing, and placement of S fertilizers on canola quality under reduced and conventional tillage. With application of plant-available forms of S fertilizer if soils were deficient in available sulphate-S, authors observed an increase in oil concentration of canola seed increased but a decrease in chlorophyll content. Therefore, authors corrected the S deficiency using fertilizer sources to improve the canola seed quality. However, the magnitude and consistency of fertilizer effects were noted to reflect the sulphate availability of the fertilizer source applied, with ammonium sulphate exhibiting a greater effect than the bentonite-elemental S product, Tiger 901, in the year of application. Although, authors further noticed inconsistent effects on seed N concentration with correction of an S deficiency but noticed occasional decreases in seed N concentration thus reflecting an inverse relationship between seed yield or seed oil concentration and seed N concentration. Moreover, authors observed a general increase in seed S concentration with increases in available S. Tillage system was found of little significance for canola quality, which was reflected by occasional reduction in oil concentration and increasing chlorophyll and seed N content. However, the response of seed quality to S fertilization was found similar both under conventional and residual tillage but sulphate-S sources were observed consistently improving the canola quality on S-deficient fields. Malik et al. [59] evaluated the influence of different levels of S fertilization (0, 25, 50, 75, 100, 125, and 150 kg ha^−1^) on seed yield in *B. napus*. Authors observed the highest seed yield (3725 kg ha^−1^) with 100 kg ha^−1^ S which was found at par with treatment where 125 kg S ha^−1^ was applied, and the minimum seed yield (2870 kg ha^−1^) was found in case of control, that is, with no S. Moreover, oil content was found progressively increased with increase of S level with highest (45.10%) with a S level of 150 kg ha^−1^. Malhi and Gill [55] conducted a 3-site-year field study to determine the response of four canola cultivars to S deficiency and S fertilization (0, 5, 10, and 15 kg S ha^−1^ rates) in terms of yield (seed and straw), seed quality (oil, protein, and S concentration), and S uptake (seed and straw) using two *B. napus* cvs. (Quantum and AC Excel) and two *B. rapa* cvs. (Maverick and AC Parkland). Authors observed that compared to *B. napus* both the actual values of seed and straw yield and seed S uptake and the responses to S fertilization were exhibited greater than *B. rapa* cvs. In addition, authors noticed an optimal yield response for all the four cultivars at the 10 kg S ha^−1^ rate, but the seed quality and S uptake were found responding up to the 15 kg S ha^−1^ rate. Moreover, S-fertilization response was observed quadratic for seed and straw yield, seed oil and protein concentration, and S uptake in seed, while authors noticed inconsistent response for seed S concentration and straw S uptake. However, authors noticed differences in magnitude of the response of tested cultivars to S fertilization, but the similar nature of the response and optimal yield at the same S rate were indicative of the fact that specific S fertilization recommendations for individual canola cultivars are unnecessary. In another study, Malhi et al. [56] studied the influence of S deficiency and S fertilization on yield, seed quality, and S uptake response of different *Brassica* oilseed species/cultivars under extensive field studies. Authors tested a total of 20 treatments in a factorial combination of four oilseed crops (*B. juncea* canola cv. Arid, *B. juncea* canola cv. Amulet, *B. juncea* mustard cv. Cutlass, and *R. napus* cv. InVigor 2663 hybrid canola) and five rates of potassium sulfate fertilizer (0, 10, 20, 30, and 40 kg S ha^−1^). With the application of 30 kg S ha^−1^, the seed yield was found maximized in all B. species/cultivars. Additionally, the oil and also protein (significant but albeit small) concentration in seed was observed increased with S fertilization for all B. species/cvs. The cv. *Cutlass juncea* mustard exhibited considerably high concentrations of glucosinolates in seed, but glucosinolate concentrations were found low in other *Brassica* species/cvs. Moreover, S-uptake in seed was noticed highest with *Cutlass juncea* mustard in all years and the effects of S deficiency and applied S were more pronounced on seed than straw. Authors concluded that all the *Brassica* species/cvs. used in this study on S-deficient soil S fertilizer require similar S for optimum seed yield, but they speculated that higher yielding types of *Brassica* species/cvs. would produce greater seed yield by using S more efficiently.

## 3. Growth, Photosynthetic Functions and Seed/Oil Yield and Quality in Oleiferous Brassicas and Nitrogen Nutrition

### 3.1. Growth and Photosynthetic Functions

The availability of N has been shown to influence the growth and photosynthetic functions to a great extent in oilseed brassicas [60–64]. Much common and growth stage specific information on N-fertilization of winter oilseed rape is available [25, 34–36, 38, 65–68]. Ogunlela et al. [62] conducted a greenhouse experiment to study N distribution and dry matter accumulation in oilseed rape (*B. napus* L. cv. *Calypso*) in relation to N supply using three levels of N supply (30, 100 or 170 ppm N). Authors observed increase in stem and leaf dry weights at higher N fertility up to 170 ppm N; they noticed no response to N by root dry weight. Dry matter yield during the vegetative phase was found seriously depressed by N deficiency. Most of the plant dry matter was found accumulated in the lower segments of the stem and roots. With the increase in N supply up to 100 ppm N, authors noticed an increase in dry weights of stem and axillary branches. Although authors observed an increase in hull dry weight increased with N supply up to 100 ppm, N they noticed no response of seed dry weight to N. Moreover, 100 or 170 ppm N maintained the high root N concentration but it was decreased with the advancing in the plant age. Furthermore, authors noted a time-depended decline in N content of leaf and stem. Leaf growth was found particularly responsive to N fertility, and N was noticed immobilized from the older to the younger leaves over time. Nitrogen content of hulls and seeds was found significantly increased with N supply, but during the pod development N was noticed translocated from the vegetative into the generative organs or from older into younger tissues. In another study, Ogunlela et al. [61] conducted an experiment in greenhouse hydroponics system to investigate the influence of N nutrition on leaf growth and chlorophyll content in oilseed rape (*B. napus* L.) during both vegetative and generative growth using three levels of N supply (30, 100 or 170 ppm N). With the N supply up to 100 ppm N to *B. napus*, authors observed increases in the leaf expansion in terms of lamina area of individual leaves and leaf area per plant, and chlorophyll content of leaves during both growth phases was increased significantly by N supply up to 100 ppm N. N supply of 30 ppm N was found creating N stress while 170 ppm N was observed as an excessive supply. Whereas, N supply of 100 ppm N was found enhancing the leaf expansion during H-6 weeks after transplanting by 88–260% over that of 30 ppm N. Moreover, authors noticed a better response of the lamina areas of younger leaves to N nutrition compared to those of older leaves. A increase of 155–194% in the leaf area per plant was noticed due to increasing N supply, but leaf number was found increased less remarkably (by 25–44%). In addition, the contents of leaf chlorophyll *a*, chlorophyll *b*, and total chlorophyll were found enhanced with N supply but resulted in very little influence on chlorophyll *a/b* ratios, except that increasing N supply tended to reduce these ratios. Authors concluded that the variation in leaf chlorophyll content of rape plants in response to N nutrition may be a function of leaf age and position which may have great significance for physiological implications. Suresh et al. [63] studied the relationship between leaf N and photosynthetic characteristics in *B. juncea*, cv. Pusa Bold and *B. campestris*, cv. Pusa Kalyani. Authors noticed a significantly higher leaf N, specific leaf weight, leaf area, and P_N_ but a significantly lower chlorophyll content in *B. juncea* compared to *B. campestris.* A significant positive correlation was obtained by the authors between leaf N content and photosynthetic rate in both species. Similarly, specific leaf weight was also found to be positively related with leaf N content. Moreover, *B. juncea* exhibited higher photosynthetic nitrogen use efficiency than *B. campestris.* Furthermore, authors noted a negative association of leaf N with photosynthetic nitrogen use efficiency which the authors attributed to a low investment of N in photosynthesis related reactions and/or partitioning of N towards compounds functionally unrelated to photosynthesis. Additionally, authors also obtained a negative relationship between specific leaf weight and photosynthetic nitrogen use efficiency. Barlog and Grzebisz [64] conducted field experiments to evaluate the effect of timing and N fertilizer application on growth dynamics of winter oilseed rape (*B. napus* L.). Comprising seven fertilization variants namely, (a) 80 (nitrophosphate NPK, NF) + 80 (calcium-ammonium nitrate, CAN); (b) 80 (calcium-ammonium nitrate, CAN) + 80 (calcium-ammonium nitrate, CAN); (c) 80 (ammonium nitrate, AN) + 80 (ammonium nitrate, AN); (d) 80 (nitrofos NPK, NF) + 50 (calcium-ammonium nitrate, CAN) + 30 (calcium nitrate, CN); (e) 80 (calcium-ammonium nitrate, CAN) + 50 (calcium-ammonium nitrate, CAN) + 30 (CN); (f) 80 (ammonium nitrate, AN) + 50 (ammonium nitrate, AN) + 30 (calcium nitrate, CN); (g) control (without N) applied in split rates at the beginning of spring regrowth (80 kg N ha^−1^), stem elongation (80 or 50) and flower buds visible stages (30), authors observed different pattern of effects on plants grown with these treatments.

In a greenhouse experiment, Kullmann et al. [60] studied the effect of N levels (30, 100 or 170 ppm N) on the concentrations and distribution of P, K, Ca, and Mg in *B. napus*. With the increasing N supply up to 100 ppm N, authors observed increased concentrations of K and Mg in leaf after the bloom stage. Leaf Ca was found decreased with increasing N nutrition, but P concentration was found unaffected. Higher P concentrations were noted in lower leaves compared to the upper leaves. Of the four nutrients analyzed for, authors observed the lowest concentration of P in the various plant parts while the highest concentration was noted with K. Moreover, Ca/Mg ratios for the hull and branches were found increased with 100 ppm N application while the ratios for the leaves, pods, and seeds were found unaffected by N nutrition. Ahmad et al. [69] conducted experiments to screen the fourteen genotypes of *B. juncea*, namely, Bio-93-22, Pusa Bahar, Pusa Basant, Bio-322-93, Vaibhav, Varuna, RML 198, Bio-589, Kranti, Bio-97-14, Bio-824, Pusa Jai Kisan, Pusa Bold, and RML for N-use efficiency by determining the nitrogen uptake efficiency, physiological nitrogen use efficiency. Authors observed a range of nitrogen efficiency (52.7–92.8) while under N-insufficient condition, seed yield varied from 1.14 t ha^−1^ to 3.21 t ha^−1^ and 2.14 t ha^−1^ to 3.33 t ha^−1^ under N-sufficient condition. Moreover, authors explained the physiological basis of this difference in terms of nitrogen uptake efficiency and physiological nitrogen use efficiency, and their relationship with the growth and yield characteristics. Authors concluded that genotype having high nitrogen uptake efficiency and high physiological nitrogen use efficiency might help in reducing the nitrogen load on soil without any penalty on the yield.

### 3.2. Seed/Oil Yield and Quality

Oilseed rape is a heavy user of N and it was estimated that the whole crop accumulates approximately 6 kg N to produce 0.1 t of seeds [38]. Narits [70] carried out field trials to evaluate the influence of nitrogen rate and application time to yield and quality of winter oilseed rape taking into account three different nitrogen rates: 120, 140, and 160 kg ha^−1^ (in active ingredient) applied either at the beginning of spring vegetation, when the main stem was 10 cm, or at start of flowering in equal portions. Authors observed that the amount of fertilizer had not as strong impact to seed yield and quality as fertilizer application time. Moreover, the highest yields of seed and raw oil could be obtained from the variant of split-N treatment (40 + 40 + 40) of 120 kg ha^−1^. Pattl et al. [71] conducted field experiments taking into account *B. juncea*, cv. Pusa Bold, and *B. campestris*, cv. Pusa Kalyani with varying levels of N supply from 0–120 kg ha^−1^. With the increasing levels of N supply, authors observed favorable modifications in the branching pattern and the number of pods produced on different order branches, in the two species. It was noticed that approximately 80% of the total seed yield was contributed by the primary and secondary branches. However, N treatment did not show significant effect on 1000 seed weight. Compared to *B. campestris*, significantly higher yield was exhibited by *B. juncea*. A linear increase in seed yield in both the species was noted with N supply up to 120 kg ha^−1^. However, authors observed a negative impact of N supply up to 120 kg ha^−1^ on partitioning of assimilates from pod wall to seed. Authors concluded that rapeseed-mustard, grown under short winter-season environment with adequate soil moisture, has the potential for higher N-fertilizer optima exceeding 120 kg ha^−1^. Field experiments were conducted by Barlóg and Grzebisz [64] to evaluate the effect of timing and N fertilizer application on seed yield of winter oilseed rape (*B. napus*). Comprising seven fertilization variants, namely, (a) 80 (nitrofos NPK, NF) + 80 (calcium-ammonium nitrate, CAN); (b) 80 (calcium-ammonium nitrate, CAN) + 80 (calcium-ammonium nitrate, CAN); (c) 80 (ammonium nitrate, AN) + 80 (ammonium nitrate, AN); (d) 80 (nitrofos NPK, NF) + 50 (calcium-ammonium nitrate, CAN) + 30 (calcium nitrate, CN); (e) 80 (calcium-ammonium nitrate, CAN) + 50 (calcium-ammonium nitrate, CAN) + 30 (CN); (f) 80 (ammonium nitrate, AN) + 50 (ammonium nitrate, AN) + 30 (calcium nitrate, CN); (g) control (without N) applied in split rates at the beginning of spring regrowth (80 kg N ha^−1^), stem elongation (80 or 50) and flower buds visible stages (30), authors observed different pattern of effects on seed yield in plants grown with these treatments. Authors noticed the highest mean seed yield (3.64 t ha^−1^) from 80(AN) + 80(AN) and 80(CAN) + 80(CAN) variants. Moreover, taking into account the mean values of 4 years, authors observed a decreased yield with the second N rate division (80 + 50 + 30).

## 4. Growth, Photosynthetic Functions and Seed/Oil Yield and Quality in Oleiferous Brassicas and Sulphur-Nitrogen Nutrition

### 4.1. Growth and Photosynthetic Functions

The assimilatory pathways of S and N have been considered functionally convergent and well coordinated as the availability of one element regulates the other [42, 46, 47] and that C assimilation pathway is closely linked to nitrate assimilation in oilseed brassicas [21, 51]. In addition, Fismes et al. [47] reported a synergistic and antagonistic relationship of S and N use efficiency in oilseed rape, respectively, at optimum rates and excessive levels of one of the elements. Moreover, S fertilization was found to improve N use efficiency to maintain a sufficient oil content and fatty acid quality of seeds [47]. Interactive effects of S and N on the growth and development of oilseed brassicas have been studied by a number of workers [43, 72, 73]. Fazili et al. [72] conducted field experiments to determine the interactive effect of S and N on growth and yield attributes of oilseed crops *B. campestris* L. (V1) and *Eruca sativa* Mill. (V2) differing in yield potential taking into account two combinations of S and N (in kg ha^−1^): 0S + 100N (−S+N; T1) and 40S + 100N (+S+N; T2). In general, authors observed significant improvement in the growth and yield attributes of both the genotypes with the combined application of S and N (+S+N) compared with N applied alone (−S+N). Moreover, out of the two combinations used by the authors, application of 40S + 100N resulted in 142, 95, 56, and 349% enhancement in biomass accumulation, leaf-area index (LAI), leaf-area duration (LAD), and photosynthetic rate, respectively, in comparison with treatment 0S + 100N in *B. campestris*. In another study, field experiments were conducted by Fazili et al. [43] to determine the interactive effect of S and N on N-accumulation, its distribution in various plant parts, and nitrogen harvest of oilseed crops, *namely*, rapeseed (*B. campestris* L. cv. Pusa Gold) and taramira (*Eruca sativa* Mill.) differing in their N-assimilation potential. In general, authors noticed a significant increase the N accumulation in both the genotypes at all the growth stages with the application of 40S + 100 N (+S+N) compared with N applied alone (−S+N). Moreover, authors argued that the application of 40S + 100 N (+S+N) could improve the N accumulation due to the improvement in the reduction of nitrate into reduced nitrogen as evident from higher nitrate reductase activity in the leaves of plants grown with both S and N, compared with N alone. It was also evidenced by the higher nitrate-N content in the leaves of plants grown with only N (−S+N) compared to those grown with both S and N (+S+N). Additionally, authors also observed increases in seed protein content and nitrogen harvest index of both the genotypes with the application of 40S + 100 N (+S+N) compared with N applied alone (−S+N). It was concluded that combined application of S along with N (+S+N) not only increased the N-accumulation, but also its mobilization towards economic sinks.

Šiaudinis and Lazauskas [73] investigated the effect of N, S, and their interaction on rape yield and its structural components were investigated at the Lithuanian Institute of Agriculture in Dotnuva during 2003–2005 on a sod gleyic (Endocalcari-Epihypogleyic Cambisol, CMg-p-w-can) light loam. The trials were arranged according to two factorial design including 3 levels of N (0, 90 and 150 kg ha^−1^) and 3 levels of S (0, 20 and 40 kg ha^−1^). Nitrogen fertilizers increased the number of secondary branches, number of pods per plant, seed yield, however, reduced 1000 seed weight. In 2003 and 2005, the highest nitrogen efficiency was obtained by applying 90 kg ha^−1^ rate, while in the year favourable for growing 2004—150 kg ha^−1^. The application of 20 kg ha^−1^ S rate had a positive effect on the number of secondary branches, number of pods per plant and seed yield. Authors positively correlated rape seed yield with the number of pods (*r* = +0.78) and also negatively correlated with 1000-seed weight (*r* = −0.85) and argued that these two important parameters might be responsible for roughly 75% of seed yield variation.

### 4.2. Seed/Oil Yield and Quality

Interactive effect of S and N on B. campestris and Eruca sativa, differing in yield potential was investigated by Fazili et al. [72] taking into account two combinations of S and N (in kg ha^−1^): 0S + 100N (−S+N; T1) and 40S + 100N (+S+N; T2). Seed yield, oil yield, biological yield, and harvest index were found improved by 141, 171, 85, and 30%, respectively, with the use of 40S + 100N (+S+N; T2) in comparison with 0S + 100N (−S+N; T1). Authors concluded that S must be included in the nutrient management package for optimum growth and yield attributes of oilseed crops. Malhi and Gill [74] conducted field experiments to study the interactive effects of N (0, 50, 100, and 150 kg N ha^−1^) and S (0, 10, 20, and 30 kg S ha^−1^) rates on yield, seed quality, and uptake of S and N in canola. Authors noticed that the absence of S application and increasing N rate the S deficiency symptoms were more pronounced and severely reduced the yield, S concentration, oil concentration, S uptake, and N uptake of seed. Moreover, when S was applied, authors observed increases in the canola yield, S concentration, S uptake and N uptake of seed as well as the yield and S uptake of straw with increasing N rate. Additionally, irrespective of S rate, fertilizer N was found to reduce oil concentration and increase in protein concentration in canola seed. Authors also observed substantial increases in yield, S uptake and N uptake of seed and straw, and total S concentration and oil concentration in seed with S fertilization whereas they did not notice consistent change in protein concentration of seed. The S-induced changes in these traits were found generally greater at higher N rates and significant N × S interaction effects were observed by the authors more frequent and pronounced for seed yield than for straw yield, indicating that the response to N rate was relatively more dependent on the S level for seed than for straw. Ahmad and Abdin [51] evaluated the interactive effect of S and N on the oil and protein contents and the fatty acid profiles of oil in the seeds of the *Brassica* genotypes, *namely*, *B. juncea* L. Czern and Coss cv. Pusa Jai Kisan (V-1) and *B. campestris* is L. (V-2) taking into account three levels of S (0, 40 and 60 kg ha^−1^) in combination with three levels of N (60, 100 and 150 kg ha^−1^) which were tested as treatments: T-1 (0S + 100N), T-2 (40S + 60N), T-3 (40S + 100N), T-4 (60S + 100N), and T-5 (60S + 150N). Authors observed enhancement in the oil content of seeds of V-1 and V-2, respectively, by 5.0–10.9% and 6.9–8.9% with the application of combined doses of S and N, when compared with application of N without S (T-1). Moreover, maximum oil content (48.1% in *B. juncea* L. Czern and Coss cv. Pusa Jai Kisan and 51.2% in *B. campestris*) was observed in treatment of T-4 (60 kg S ha^−1^ and 100 kg N ha^−1^). Additionally, authors noticed increases in the oleic acid and linoleic acid contents and decreases in the eicosenoic acid and erucic acid contents in both genotypes with the application of S with N, when compared with N alone. Treatment (T-3) (40S + 100N) resulted in maximum contents on protein, N. and S. Authors concluded that a balanced N and S supply should be maintained for both quantity and quality of oil of *Brassica* genotypes. Jan et al. [75] evaluated the effects of N and S levels and their methods of application on quality parameters of canola (*B. napus* L. cv. “Bulbul-98”) taking into account four levels of S (0, 20, 40, and 60 kg ha^−1^) and three levels of N (80, 120, and 160 kg ha^−1^) and a control treatment (with both nutrients at zero level) applied either as a sole dose at sowing, in two split applications (half each at sowing and leaf rosette stages) or three split applications (one third each at sowing, leaf rosette stage, and early flowering). Authors observed large increases in oil and protein concentrations at 40 kg S ha^−1^ while they did not notice further significant increase with increasing S level (60 kg S ha^−1^). However, authors observed consistent increase in glucosinolate concentrations with the highest level of 60 kg S ha^−1^. The application of 160 kg N ha^−1^ resulted in significant increase in protein concentrations while glucosinolate concentrations were found increased up to 120 kg N ha^−1^. Moreover, oil concentrations were exhibited a negative trend to increasing N level.

### 4.3. Sulphur-Nitrogen Interaction and N/S-Use Efficiency

As stated also above that N and S nutrition are tightly linked during the growth cycle [42, 47], Fismes et al. [47] reported that the S and N use efficiency of oilseed rape is synergistic at optimum rates and antagonistic at excessive levels of one of the elements. S fertilization is required to improve N use efficiency and thereby maintain a sufficient oil content and fatty acid quality of seeds [47]. Additionally, S and N relationship in terms of crop yield and quality has been established in many studies [12, 47, 53, 76–78]. Moreover, the combined application of S and N promotes the uptake of S and N, which lead to significant enhancement in seed protein and oil content in *B. juncea* and *B. campestris* [12, 21, 51, 79]. Sulphur and N relationship in terms of crop yield and quality has been established in many studies [12, 47, 53, 76–78]. The impacts of S deprivation on seed quality and yield have been shown to depend on N supply [44]. In addition, S availability may influence N use efficiency (NUE) of oilseed rape and vice versa [46, 47], indicating that mineral S and N availabilities closely interact with S and N management by the plant [80]. Abdallah et al. [28] conducted a study to determine the effects of mineral S limitation on N and S uptake and remobilization during vegetative growth of oilseed rape at both the whole-plant and leaf rank level for plants grown during 35 d with 300 *μ*M ^34^(SO_4_^2−^) (control plants; +S) or with 15 *μ*M ^34^(SO_4_^2−^) (S-limited plants; −S). Authors observed no significant differences either in whole-plant and leaf biomass or in N uptake in S-limited plants when compared with control plants. However, authors noted great reduction in total S and S-34 (i.e., deriving from S uptake) contents for the whole plant and leaf after 35 d; a greater redistribution of endogenous S from leaves to the benefit of roots was also observed. Authors concluded that (a) S-limitation in oilseed rape does not change its development despite the 20-times less mineral S compared to control plants and (b) endogenous S compounds (mostly sulphate) are recycled from leaves to roots during S limitation in oilseed rape.

## 5. Sulphur and Nitrogen Nutrition and Their Interaction-Mediated General Defense Mechanisms against Stresses: Overview

Oxidative stress is a central factor in abiotic stress phenomena that occurs when there is a serious imbalance between the production of reactive oxygen species (ROS) and antioxidant defense in different cellular compartments including chloroplasts, mitochondria, and peroxisomes, leading to dramatic physiological changes [81–83]. ROS-lead oxidative stress, in general, has been shown to trigger mainly the peroxidation of membrane lipids, and consequent severe damages to biological molecules including nucleic acids, lipids and proteins and/or cell death. Therefore, an equilibrium between the production and elimination of ROS must be maintained in cells if metabolic disorder or oxidative burst is to be avoided [81, 83, 84]. Any adaptation that regulates ROS generation in plants will provide efficient defense mechanism for tolerance against stress.

In plants, the ascorbate-glutathione (AsA-GSH) pathway has been extensively evidenced as the central component of plant antioxidant defense system to efficiently remove/metabolize ROS and/or their reaction products; hence, AsA-GSH pathway is important for the maintenance of cellular homeostasis in plants under variety of stress conditions. Most importantly, among the major components of AsA-GSH pathway and as an important nonenzymatic antioxidant/metabolite, tripeptide glutathione (GSH, *γ*-glutamate-cysteine-glycine) is considered as the most important intracellular defense against ROS- and/or their reaction products-induced oxidative damage in plants [17, 83, 85, 86].

Among plant nutrients, S has been reported as the major modulator of GSH-mediated control of plant stress tolerance [87, 88]. Sulfur is incorporated into organic molecules in plants and is located in thiol (–SH) groups in proteins (cysteine/Cys-residues) or nonprotein thiols (NPT; GSH), maintains homeostasis of GSH and oxidized glutathione (GSSG) ratios, and protects plants from oxidative damage. Glutathione plays a multifaceted role in plant metabolism. Plants can withstand Cd toxicity by maintaining high levels of phytochelatin (PC) or its precursor, GSH, which functions as a metals ligand.

As mentioned also above that the requirement of S and its metabolism in plants including oleiferous brassicas are closely related to N nutrition [42, 43], and where N metabolism has been shown to be strongly influenced by the S status of the plant [43–45], the assimilation pathways of both S and N have been reported very similar and well coordinated and convergent [42, 79, 89–91]. Deficiency of one element was shown to repress the other pathway [9, 47, 92–94]. Additionally, a positive interaction between S and N has been reported to be beneficial for various aspects of oilseed brassicas including tolerance to various stress factors.

**Figure 1:**
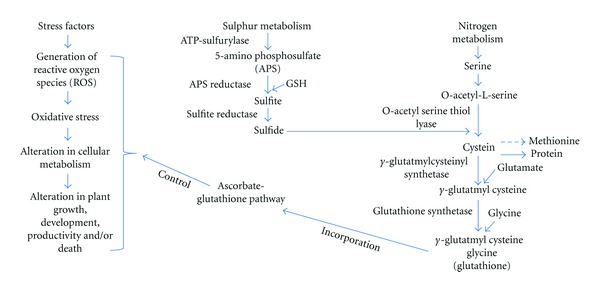
A schematic presentation of major events in sulfur (S) and nitrogen (N) nutrition-mediated control of glutathione (GSH) biosynthesis and its subsequent incorporation into ascorbate-glutathione (AsA-GSH) pathway for the regulation of cellular metabolism, plant growth, development, and productivity *via* removal and/or metabolism of stress-induced/generated reactive oxygen species (ROS) in plants. *See text for details. *

In the context of S-N-mediated control of plant stress tolerance, the biosynthesis of GSH depends on the availability of its constituent amino acids, where glutamate (Glu) is provided through assimilation of N, an essential component in agricultural production, while Cys is shared by reductive assimilation of S as the end product. The availability of Glu and Cys (and GSH), thus, may be influenced with N and S supply, respectively. A number of major plant stress-defense operations have been reported to be significantly enhanced by supplementary S fertilization to high S loving crops such as Brassicas and leguminous crops. In fact, S supplementation indirectly improves general plant performance under abiotic and biotic stresses by improving AsA and GSH [95–97]. Anjum et al. [96] observed interesting relationships between AsA and GSH pools with net photosynthesis and plant dry mass with and without-S in *Brassica campestris*. Authors suggest that adequate S supply may improve the pools of these compounds in plants to a great extent that may lead to increase in photosynthetic efficiency and subsequently to plant dry mass and crop yield. Sufficient S supply was reported to improve photosynthesis and growth of *B. juncea* through regulating N assimilation [98]. Moreover, Cobbett [99] and Leustek et al. [15] reported the close dependency of PCs biosynthesis on S metabolism. Expression of genes involved in reductive sulphate assimilation pathway and enzyme activities are stimulated by cadmium [100, 101]. Cadmium exposure induces the activity of enzymes (*γ*-glutamyl-Cys synthetase, *γ*ECS and glutathione synthetase, GS) involved in the biosynthesis of GSH. Herbette et al. [100] suggested that plants activate the S assimilation pathway by increasing transcription of related genes to provide an enhanced supply of GSH for PC biosynthesis to cope with cadmium toxicity. GSH has been shown to contain three moles of N per mole of S and that the biosynthesis of GSH may depend on the availability of N precursors and thus N nutrition of plants. However, the sink strength of GSH biosynthesis for N may be low compared with the other major N sinks such as the synthesis of proteins or nucleotides [80]. The GSH biosynthesis is, therefore, regulated not only by the dependency of Cys availability on S, N, and C metabolism, but also on the availability of Glu and Gly. In this way, the coordinative functions of S and N may strengthen the stress tolerance ability of plants growing under abiotic stresses. A schematic presentation of major events in S-N nutrition-mediated control of GSH, GSH/GSSG ratio for the removal and/or metabolism of stress-induced/generated ROS in plants (hence, the control of plant growth, development and productivity) has been depicted here in the current paper (Figure 1).

## 6. Conclusions

Oleiferous Brassicas (rapeseed and mustard) are among important food crops in Asia. In India, the oilseeds form the second largest agricultural commodity where among nine annual oilseed crops grown, the oleiferous Brassicas are the major provider of edible oil to a major proportion of population. Among the oleiferous Brassicas, rapeseed and mustard are important species grown as oilseeds. These have remained one of the major sources of edible for centuries. In view of increasing population and improved and increased standard of living of the people in 21st century, the requirement of fats and oils is bound to go high. To meet the minimal nutritional requirement of fats and oils (12 kg capita^−1^ year^−1^) for food, feed and other industries, and to earn sizable foreign exchange through export of seed meal, oil and value added products, nearly 24 million tonnes of mustard oilseed would be required by 2020 AD. Moreover, since last few decades, the growth, development and productivity of oleiferous brassicas have been hampered due to a number of factors including the unbalanced plant mineral nutrients in soils. The plant nutrients, S and N, are of great importance. Sulphur is an essential macronutrient of plants that plays a vital role in the regulation of plant growth and development. In addition, S is a structural constituent of several coenzymes and prosthetic groups, such as ferredoxin, which is also important for N assimilation. Nitrogen is another important component of several important structural, genetic, and metabolic compounds in plant cells. Plant N status is highly dependent on N fertilization. N is also a major component of chlorophyll and amino acids, the building blocks of proteins. Increase in N supply can stimulate plant growth and productivity, as well as photosynthetic activity through increased amounts of stromal and thylakoid proteins in leaves. Moreover, oilseed brassicas require higher amounts of S in addition to N for optimum growth and yield. Several studies have established positive interaction between S and N which was found to be beneficial for various aspects of oleiferous brassicas. Therefore, managing the optimum level of S and N mineral nutrients in plants and soils may result into improved growth, development, and productivity in S-N-loving oleiferous brassicas.

The biosynthesis of GSH (a major component of AsA-GSH pathway and as an important nonenzymatic antioxidant/metabolite, considered as the most important intracellular defense against ROS- and/or their reaction products-induced oxidative damage in plants) depends on the availability of its constituent amino acids, where Glu is provided through assimilation of N, an essential component in agricultural production, while Cys is shared by reductive assimilation of S as the end product. The availability of Glu and Cys (and GSH), thus, may be influenced with N and S supply, respectively. A number of major plant stress-defense operations have been reported to be significantly enhanced by supplementary S fertilization to high S-loving crops such as Brassicas and leguminous crops. Thus, S-N nutrition and their interaction studies may provide a novel strategy to reduce the adverse stress effects through increased N utilization and synthesis of reduced S compounds including Cys and GSH in economically important crop plants under changing environment.
